# Effects of Sprint Interval Training on Brain Fatigue Resistance in Competitive Skateboarders: Evidence from EEG, HRV, and VAS Measures

**DOI:** 10.3390/life16010025

**Published:** 2025-12-24

**Authors:** Mulin Yang, Yuqiang Guo, Kewei Zhao

**Affiliations:** 1 Physical Fitness Training Research Center, China Institute of Sport Science, Beijing 100061, China; yanis11011@163.com; 2 School of Physical Education, Shanghai University of Sport, Shanghai 200438, China; guoyuqiang824@163.com

**Keywords:** cognitive fatigue, sprint interval training, executive function, neurocognitive adaptation, fatigue monitoring techniques

## Abstract

Purpose: This preliminary study examined the associations between a 6-week sprint interval training (SIT) program and mental-fatigue (MF) related neurophysiological and subjective indicators in elite skateboarders. Methods: In this preliminary study, a single-group, repeated-measures design was employed. Twelve elite skateboarders completed a 6-week sprint interval training (SIT) program. Mental fatigue was assessed at three time points—pre-intervention (Week 0), mid-intervention (Week 3), and post-intervention (Week 6)—using a standardized 60 min Stroop task, with post-task EEG, HRV, and VAS measures collected to characterize neurophysiological and subjective responses. Results: Across the intervention, EEG indices indicated higher central nervous system activation and more stable post-task neural profiles. HRV indices suggested more flexible autonomic regulation, with favorable changes in low- and high-frequency components, sympathovagal balance, and recovery-related scores, whereas baseline-related indices such as RMSSD and SDNN showed no clear change. VAS ratings showed stable MF, accompanied by increased mental exertion and motivation and reduced physical fatigue over time. Conclusions: These preliminary findings suggest that a 6-week SIT program may be associated with enhanced resistance to Stroop-related MF in elite skateboarders, potentially through coordinated adaptations in neural activation, autonomic regulation, and psychological factors. Future randomized studies incorporating behavioral performance and sport-specific cognitive tasks are warranted to confirm and extend these observations.

## 1. Introduction

Recentreports estimate that over 85 million people worldwide participate in skateboarding recreationally or competitively [1]. The sport has grown rapidly following its Olympic debut at Tokyo 2020 and continued inclusion in Paris 2024 and Los Angeles 2028 [1,2]. Skateboarding has developed into a global youth-oriented sport that integrates creativity, technical complexity, and high-risk performance [3]. The sport is officially divided into Street and Park disciplines, which differ in structural layout and trick composition but both require exceptional coordination, balance, and reaction speed [4,5]. In elite competitions, skateboarders must rapidly process sensory information, make split-second decisions, and execute technically complex movements under time pressure and psychological stress [6]. These cognitive demands increase the risk of mental fatigue (MF)—a neuropsychological state characterized by reduced attention, slower reaction times, and impaired decision-making after prolonged cognitive effort [7]. MF not only impairs perceptual-cognitive abilities but also disrupts neuromuscular signaling, reduces motor coordination, and induces negative emotional states such as anxiety and frustration [8]. This creates a vicious cycle of “cognitive overload—movement errors—emotional instability” that undermines performance. Previous research has primarily addressed injury epidemiology [5,9], biomechanics [3], and physiological demands [10], whereas mental and cognitive performance factors have received comparatively little attention. Although MF has been studied in endurance and team sports, its implications for high-skill, high-risk, creativity-driven sports such as skateboarding remain under explored, warranting further empirical investigation.

Current research [2,11] on skateboarder training has focused mainly on technical and tactical aspects, whereas empirical studies addressing cognitive and fatigue-related effects of training remain scarce. Against this background, High-Intensity Interval Training (HIIT), particularly Sprint Interval Training (SIT), has been widely applied to improve athletes’ physical fitness and neuromuscular regulation [12,13,14]. SIT generally consists of repeated 15–45 s maximal sprints followed by 3–5 min of full recovery. This format rapidly induces maximal blood lactate accumulation, strongly stimulates both neuromuscular and anaerobic glycolytic systems, and effectively improves anaerobic power and recovery efficiency [15]. Because skateboarding emphasizes explosive output, rapid recovery, and continuous perceptual–cognitive control under pressure, its movement and decision-making characteristics align with the physiological and neuro-regulatory demands targeted by SIT. This makes SIT a theoretically suitable modality not only for improving performance consistency, but also for enhancing resistance to mentally fatiguing cognitive load during competition.

In addition to its established physiological benefits, emerging evidence suggests that SIT may also influence brain function through several neurobiological mechanisms. Briefly, SIT has been shown to increase brain-derived neurotrophic factor, enhance antioxidant defenses and mitochondrial biogenesis, and promote lactate production and utilization in the brain, with lactate acting as both an energy substrate and a neuromodulator for dopaminergic and cholinergic signaling related to attention, learning, and executive control [16,17,18,19,20,21]. Collectively, these adaptations may help the brain maintain stable function under prolonged cognitive load and could, in turn, contribute to greater resistance to mental fatigue in cognitively demanding sports such as skateboarding. However, these mechanistic pathways were not directly assessed in the present study and are presented here only as conceptual background. Although research on the cognitive effects of HIIT and SIT has advanced in recent years, most studies have focused on general populations [22,23] or endurance sports [24], with little attention to high-skill, cognitively demanding disciplines such as skateboarding. Existing work has largely emphasized cardiorespiratory and physical performance outcomes, whereas systematic evaluations of SIT in relation to mental fatigue resistance and neuro-regulatory adaptation in elite skateboarders are lacking.

Taken together, current research on skateboarding has emphasized biomechanics, injury epidemiology, and physical conditioning, while the neurocognitive consequences of training interventions have received limited attention [3,5,9,10,22,23,24]. Likewise, although the cognitive effects of HIIT and SIT have been explored in general and endurance populations, little is known about how SIT relates to mental fatigue resistance and neuro-regulatory adaptation in elite athletes participating in high-skill, cognitively demanding sports such as skateboarding. This gap motivates the present study.

Against this background, we framed the study within three complementary sport psychology frameworks to guide our rationale and interpretation. The Psychomotor Efficiency Hypothesis states that high-level motor performance reflects more economical cortical regulation with less unnecessary neural interference [25]. The Processing Efficiency Theory distinguishes between performance effectiveness and the cognitive cost required to sustain performance [26]. Thus, stable outcomes can occur even when perceived effort changes. The Multi-Action Plan model further conceptualizes performance states along a continuum ranging from automatic and effective control to effortful and controlled modes [27,28,29]. Accordingly, we anticipated that SIT would not only influence performance-related capacity, but would also alter the style of neural control toward a more efficient and automatic mode, which may contribute to greater resistance to mentally fatiguing cognitive load in elite skateboarders.

Given skateboarding’s explosive, technical, and cognitively demanding nature, athletes require strong resistance to brain fatigue to maintain optimal performance. With its multifaceted benefits in energy metabolism and neural regulation, SIT provides a theoretical basis for enhancing neurocognitive adaptation in this population. Therefore, investigating SIT’s effects on MF resistance in elite skateboarders is both theoretically relevant and practically significant, supporting the development of more scientifically grounded high-performance training systems.

Therefore, the present study aimed to examine how a 6-week SIT program relates to mental fatigue resistance in elite skateboarders by tracking EEG, HRV, and VAS responses to a standardized Stroop task at pre-, mid-, and post-intervention. Based on the above rationale, we hypothesized that: (1) EEG indices would show enhanced central nervous system activation and greater post-task stability over time; (2) HRV indices would reflect more flexible autonomic regulation, with improved sympathovagal balance and recovery-related markers; and (3) subjective VAS ratings would indicate stable mental fatigue alongside increased mental exertion and motivation and reduced physical fatigue following the cognitive task.

## 2. Materials and Methods

### 2.1. Research Design

This preliminary study employed a single-group, repeated-measures design conducted within the centralized training environment of the Chinese national skateboarding team. The primary aim was to explore within-athlete changes in resistance to Stroop induced mental fatigue following a 6-week SIT program. Assessments were performed at three time points: pre-intervention (Week 0), mid-intervention (Week 3), and post-intervention (Week 6). Because competitive skateboarding has only recently become an Olympic discipline, the number of elite athletes who meet national team selection and health criteria is structurally limited, and all squad members follow a unified high performance training schedule. Under these practical constraints, randomization and the inclusion of a parallel control group were not feasible. Instead, all eligible athletes were enrolled in the SIT program, and within-subject changes over time were used to characterize preliminary neurophysiological and psychological adaptations.

### 2.2. Participants

Twelve elite skateboarders (7 males, 5 females; age: 16.2 ± 2.8 years; height: 163.2 ± 8.5 cm; weight: 52.0 ± 7.8 kg) from the Chinese national skateboarding team volunteered to participate in this preliminary study. Participants were required to meet the following criteria: (1) Official international rankings within the past three years; (2) at least five years of structured skateboarding-specific and physical training experience; (3) no systematic SIT within the past six months; (4) healthy, with normal vision and no cardiovascular or neuromuscular disorders affecting high-intensity tests; (5) no insomnia, medication dependence, or unhealthy habits such as smoking or excessive alcohol consumption.

All participants and their parents or legal guardians provided written informed consent, and none withdrew from the study. The study was conducted in accordance with the Declaration of Helsinki and was approved by the Ethics Review Committee of the General Administration of Sport of China (approval No. 20250722).

### 2.3. Experimental Procedures

This preliminary study examined the associations between a 6-week SIT intervention and MF related neurophysiological and subjective indices in elite skateboarders. The intervention was conducted from July 13 to 23 August 2025, with three weekly sessions integrated into routine team conditioning and technical training (18 sessions total). The content and overall intensity of the team’s regular conditioning and technical sessions were kept consistent with the pre-intervention period. No additional high-intensity training was introduced beyond the prescribed SIT protocol. Participants were instructed to maintain habitual training volume and intensity, and compliance was verified via weekly logs and coach supervision.

Each training session lasted approximately 45 min and consisted of: (1) A 15 min dynamic warm-up to activate major muscle groups and increase heart rate; (2) six 30 s maximal climbing sprints on a vertical climbing ergometer (indoor climbing machine; Model VC-1000, Shandong FitClimb Sports Equipment Co., Ltd., Shandong, China; difficulty levels 16–25), with training intensity maintained at 85–95% HRmax, continuously monitored in real time using a Polar heart-rate chest strap (Polar H10, Polar Electro Oy, Kempele, Finland), interspersed with 150 s passive or low-intensity active recovery intervals between sprints [13]; (3) 5 min of low-intensity climbing or cycling as post-session active recovery, followed by static stretching focusing on lower limbs and core musculature. During this phase, athletes were instructed to maintain contralateral limb coordination to avoid compensatory movement patterns and were closely monitored for signs of excessive physiological strain (e.g., dizziness, nausea) that would prompt immediate termination. Ratings of perceived exertion (RPE; Borg 6–20 scale) and training completion rates were recorded to ensure intervention fidelity [30].

The climbing machine was selected in the study because it requires multi-joint coordination similar to skateboarding [11,31] and effectively develops the dynamic balance and core strength required for skateboarding performance [14]. Furthermore, evidence has shown that 30 s SIT can typically elicit heart rates approaching 85–95% of HRmax, and this phenomenon has been consistently demonstrated across SIT protocols [32]. Within the constraints of the indoor national training facility, repeated maximal sprint-running or skateboard-based sprinting at comparable intensity was not feasible from a safety and logistical standpoint. All training sessions were supervised throughout by certified strength and conditioning coaches and exercise physiology experts.

Tests were conducted at three time points—pre-intervention (Week 0), mid-intervention (Week 3), and post-intervention (Week 6)—all scheduled on Sunday mornings (rest days). At each time point, participants completed the following tasks: (1) Visual Analogue Scale (VAS) assessments of MF, mental exertion (ME), motivation (MO), and physical fatigue (PF); (2) Stroop MF induction tasks; and (3) repeated VAS assessments and OmegaWave electroencephalography/heart rate variability (EEG/HRV) system data collection. After completing the Stroop task, participants sat quietly in a seated resting position for at least 5 min, and EEG/HRV recordings were obtained during this post-task rest period. This 60 min incongruent Stroop protocol has been widely used and validated to induce sustained mental fatigue in competitive athletes [33]. Specifically, four Chinese characters (“red”, “green”, “blue”, “yellow”) in Song font (size 57) were presented sequentially in random order against a black background. The font color of each character (red, green, blue, or yellow) had a 50% probability of mismatching its semantic meaning. Participants identified the font color by pressing the corresponding key: for green, blue, or yellow, they pressed the key matching the font color (ignoring the character’s meaning); for red, they pressed the key corresponding to its semantic meaning. Each character was displayed for 1000 ms, followed by a 1000 ms blank screen, resulting in a total trial duration of 2000 ms. The 60 min Stroop task consisted of four blocks of 450 trials (15 min each), totaling 1800 trials, with 30 s intervals between blocks for data collection. Before the task, participants completed 20 practice trials to ensure understanding of the response requirements. During the task, participants were instructed to respond as quickly and accurately as possible, with errors or delays indicated by an auditory beep. Testing was conducted in a quiet environment with appropriate lighting, where participants maintained a normal sitting posture without distractions. All tests were conducted at the same time of day to minimize circadian rhythm interference, with standardized warm-ups preceding each test. In this experiment, the Stroop task was employed solely as a mental fatigue induction paradigm, and reaction time and accuracy were not analyzed; instead, EEG, HRV, and VAS were used to quantify adaptations in resistance to Stroop-induced mental fatigue.

To control external factors, participants were prohibited from engaging in additional high-intensity training during the 6-week experiment, beyond their standard team conditioning sessions and the prescribed SIT protocol and were instructed to maintain adequate sleep and a balanced diet before each test and training session while avoiding stimulant-containing beverages. The entire experiment was supervised by certified strength and conditioning coaches and exercise physiology experts.

Pre-task EEG and HRV measurements were not included to avoid extending the testing session and introducing additional cognitive or physiological load prior to the mental fatigue induction. In an elite training context, minimizing pre-task burden was considered important to preserve ecological validity and participant compliance. Post-task recovery measures were therefore prioritized as they were deemed more relevant for capturing regulatory stability and fatigue-related adaptation following sustained cognitive stress.

### 2.4. Testing Procedures

#### 2.4.1. Electroencephalography (EEG)

EEG data were collected using the OmegaWave device (Model OW-CB2, Omegawave Oy, Espoo, Finland; software version 3.3.423). To ensure accuracy and reliability, raw EEG signals were preprocessed with standardized procedures, including noise filtering, artifact correction, and outlier removal. Power spectral density of specific frequency bands (e.g., α and β waves) was extracted as indicators of brain activity under different functional states. Table 1 summarizes EEG indicators across multiple dimensions, reflecting brain adaptation and recovery during high-intensity exercise. CNS activation level, DC stabilization time, and stabilization level indicate post-exercise recovery speed and regulatory capacity, while DC tension and exhaustion level represent physiological and psychological load, providing insight into the athletes’ fatigue resistance potential.

#### 2.4.2. Heart Rate Variability (HRV)

HRV data were collected using the OmegaWave system (Omegawave, Espoo, Finland). To ensure accuracy and stability, environmental interference was minimized, and participants remained in a seated resting state during a standardized post-Stroop recovery period while recordings were obtained. HRV analysis (Table 2) included time-domain indices (e.g., SDNN, RMSSD) and frequency-domain indices (e.g., LF, HF) to evaluate autonomic nervous system (ANS) regulation and function. Beyond these indices, the OmegaWave system generated extended parameters, including PNS (parasympathetic nervous system score), SNS (sympathetic nervous system score), Ti (tension index), and Recovery Pattern. These indices are closely related to neural recovery, fatigue perception, and adaptation following high-intensity training or cognitive tasks, making them widely applied in the dynamic monitoring of training load and recovery in athletes. HRV indices reflect sympathetic parasympathetic balance, assess stress and recovery after SIT or other high-intensity interventions, and quantify self-regulation capacity after fatigue through RMSSD and SDNN. As a reliable, non-invasive physiological tool, HRV provided objective evidence to evaluate SIT’s impact on neural regulation and fatigue recovery in elite skateboarders. Although some components of OmegaWave’s algorithmic modeling are proprietary, all raw EEG and HRV signals underwent standardized preprocessing (noise filtering, artifact correction, and outlier removal). Future research should incorporate open-source analytic pipelines and additional physiological markers to further enhance reproducibility.

#### 2.4.3. Subjective Measurement

MF was evaluated using a 100 mm VAS, a simple and widely applied tool with established reliability, validity, and sensitivity for assessing psychophysiological states in elite athletes [34,35]. For each 100 mm scale, the left anchor (0 mm) was labeled “not at all” and the right anchor (100 mm) was labeled “extremely” for the corresponding dimension (e.g., “not at all mentally fatigued” to “extremely mentally fatigued” for MF). Before the Stroop task, participants rated their current mental states, including MF, mental exertion (ME), motivation (MO), and physical fatigue (PF). After baseline assessment, they completed a 60 min Stroop task in random balanced order, followed by reassessment with the same VAS. Differences between pre- and post-task scores reflected Stroop-induced MF and the potential modulatory effect of SIT on MF recovery.

### 2.5. Statistical Analyses

All statistical analyses were performed using IBM SPSS Statistics 27.0 software. Given the small sample size (*n* = 12), the Shapiro–Wilk test (*n* < 50) was used to examine the normality of the data for each variable at the pre-, mid-, and post-intervention stages, as this method is considered more appropriate for small samples. Based on the results of the normality test, different analytical approaches were applied: (1) If the data met the normality assumption, Mauchly’s test of sphericity was first conducted. When the assumption of sphericity was violated, the Greenhouse–Geisser correction was applied. A one-way repeated-measures analysis of variance (ANOVA) was then used to compare the differences among the three time points. Post hoc pairwise comparisons were adjusted using the Bonferroni correction to control for Type I error inflation [36]. The effect size was reported as partial *η*^2^, with interpretation thresholds defined as small (0.01 ≤ *η*^2^ < 0.06), medium (0.06 ≤ *η*^2^ < 0.14), and large (*η*^2^ ≥ 0.14) according to Cohen (1988) [37]. (2) If the data did not meet the normality assumption, the non-parametric Friedman test was employed to compare the pre-, mid-, and post-intervention results. When a significant difference was found, pairwise comparisons were conducted using the Wilcoxon signed-rank test with Bonferroni adjustment to control for multiple testing. The effect size was reported as Kendall’s W, and interpreted as small (0.10 ≤ *W* < 0.30), medium (0.30 ≤ *W* < 0.50), and large (*W* ≥ 0.5) according to Fritz (2012) [38]. All data are presented as mean ± SD, and the level of statistical significance was set at *p* < 0.05. Given the small sample size and within-subject repeated-measures design, no covariates (e.g., sex, age, training experience) were entered into the models to avoid overfitting and unstable parameter estimates. Effect sizes were reported to facilitate the interpretation of practical relevance given the small sample size and exploratory nature of the study.

## 3. Results

Given the preliminary single-group repeated-measures design, results are presented as within-athlete changes across the three assessment time points rather than as between-group comparisons. Accordingly, the findings should be interpreted as exploratory patterns of association over time.

### 3.1. EEG

**Figure 1:**
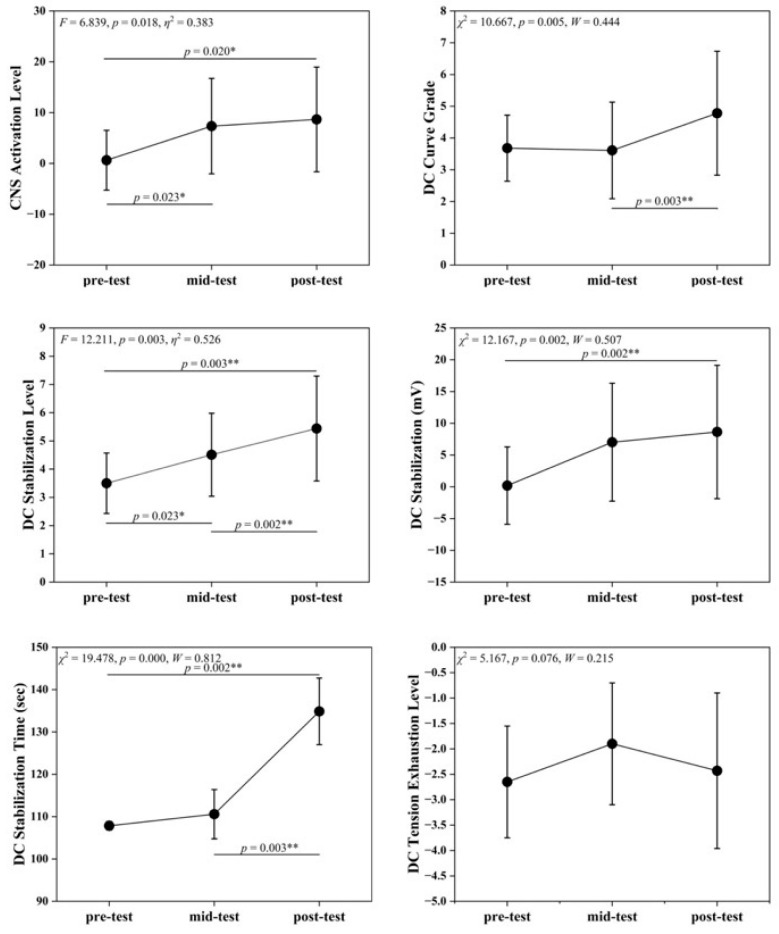
Changes in EEG Indicators across pre-test, mid-test, and post-test. Symbols denote post hoc comparisons. The asterisk (*) indicates a statistically significant difference, and the double asterisks (**) indicate a highly statistically significant difference.

Table 3 and Figure 1 illustrate significant time effects in EEG indices across the pre-intervention, mid-intervention, and post-intervention phases. CNS Activation Level differed significantly (*F* = 6.839, *p* = 0.018), with mid- and post-intervention values higher than pre-intervention (*p* < 0.05), which tentatively suggests that SIT may enhance central nervous system activation. DC Curve Grade showed highly significant differences (*χ*^2^ = 10.667, *p* = 0.005), with post-intervention > mid-intervention (*p* < 0.01), indicating improved neural responsiveness to physiological and psychological load changes. DC Stabilization Level and DC Stabilization differed significantly across time points (*F* = 12.211, *χ*^2^ = 12.167, *p* < 0.01). DC Stabilization Level increased progressively, with significant differences between all time points (*p* < 0.05). DC Stabilization showed highly significant differences between pre- and post-intervention (*p* < 0.01). These findings support the interpretation of faster post-training neural stabilization and reduced signal fluctuations. DC Stabilization Time differed significantly (*χ*^2^ = 19.478, *p* < 0.001), with post-intervention values longer than previous measurements, which may reflect increased regulatory load alongside enhanced neural activation. DC Tension Exhaustion Level showed a non-significant trend (*χ*^2^ = 5.167, *p* = 0.076).

### 3.2. HRV

**Figure 2:**
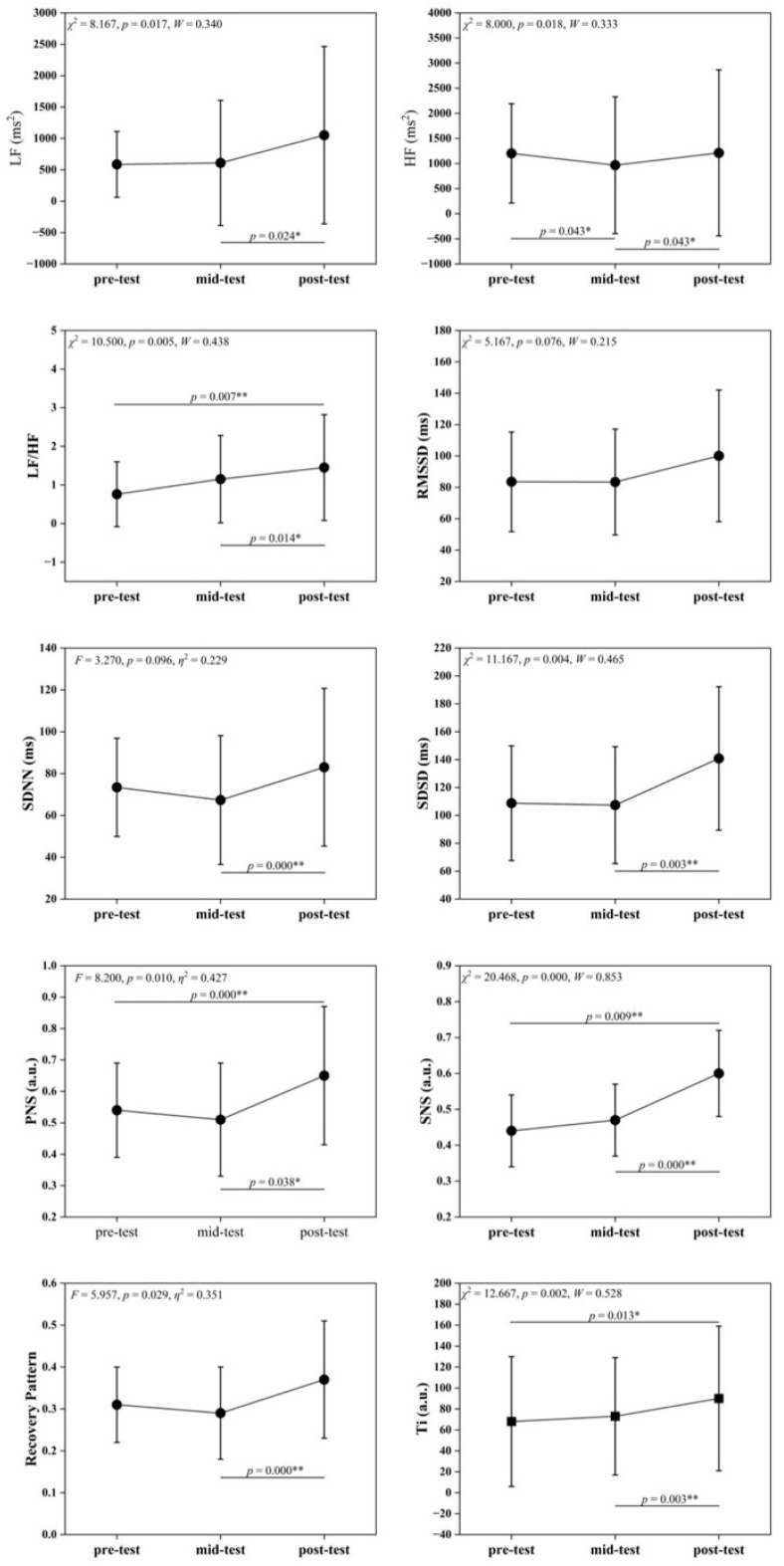
Changes in HRV Indicators across pre-test, mid-test, and post-test. Symbols denote post hoc comparisons. The asterisk (*) indicates a statistically significant difference, and the double asterisks (**) indicate a highly statistically significant difference.

Table 4 and Figure 2 illustrate significant differences in multiple HRV indices across time points. LF values differed significantly (*F* = 8.167, *p* = 0.017), with post-intervention > mid-intervention (*p* < 0.05), suggesting enhanced sympathetic activity. HF values differed significantly (F = 8.000, *p* = 0.018), with pre- and post-intervention > mid-intervention (*p* < 0.05), suggesting that parasympathetic activity temporarily decreased and subsequently recovered. The LF/HF ratio differed highly significantly (*F* = 10.500, *p* = 0.005), showing an upward trend that indicated an adjusted autonomic balance. SDSD, PNS, and SNS indices differed significantly (all *p* ≤ 0.05), with higher post-intervention values suggesting enhanced autonomic regulation and improved sympathetic parasympathetic activity. RP and Ti changed significantly (*F* = 5.957, *p* = 0.029; *χ*^2^ = 12.667, *p* = 0.002). RP increased at post-intervention, while Ti rose progressively, suggesting that SIT improved recovery patterns and adaptation to training stress. In contrast, time-domain indices such as RMSSD and SDNN did not show statistically significant main time effects (both *p* > 0.05), indicating that the observed autonomic adaptations were primarily reflected in specific frequency-domain and composite regulatory markers.

### 3.3. VAS

**Figure 3:**
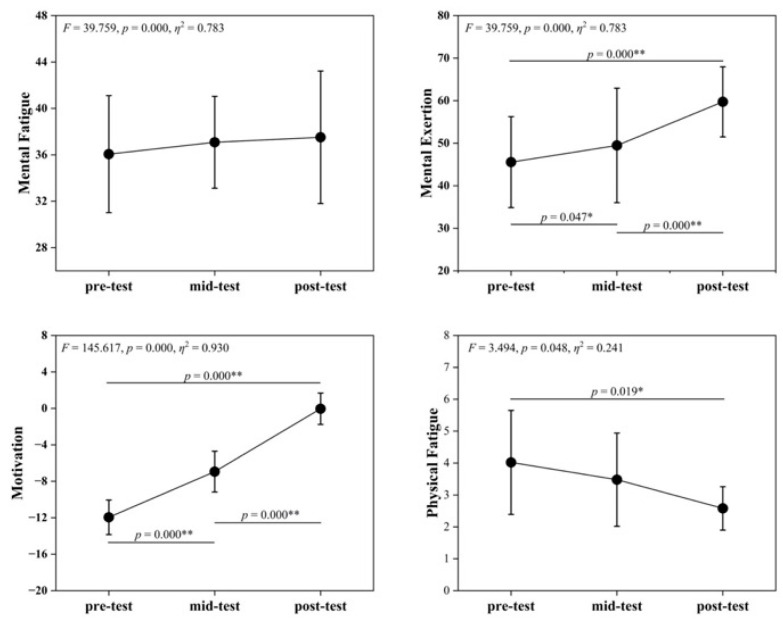
Changes in VAS Indicators across pre-test, mid-test, and post-test. Symbols denote post hoc comparisons. The asterisk (*) indicates a statistically significant difference, and the double asterisks (**) indicate a highly statistically significant difference.

Table 5 and Figure 3 show no significant differences in MF scores across time points (*F* = 1.156, *p* = 0.333), indicating that SIT had limited immediate effects on subjective MF. ME differed significantly (*F* = 39.759, *p* < 0.001), with post-intervention > pre- and mid-intervention, potentially reflecting increased perceived cognitive resource investment. MO also changed highly significantly (*F* = 145.617, *p* < 0.001), showing an upward trend that suggested enhanced task motivation, possibly due to familiarity or training incentives. PF differed significantly (*F* = 3.494, *p* = 0.048), with post-intervention < pre-intervention, indicating improved recovery from physical fatigue.

## 4. Discussion

This study investigated neurophysiological and psychological adaptations to a 6-week SIT program in elite skateboarders, with a particular focus on MF resistance assessed through EEG, HRV, and VAS measures. Results indicated that SIT was associated with increased CNS activation and stability, more favorable autonomic nervous system regulation, and improved subjective indicators of motivation and physical fatigue. Although subjective MF remained stable across time points, the combined neurophysiological and psychological adaptations provide preliminary evidence that SIT may strengthen multi-system resistance to cognitive fatigue in high-performance skateboarding.

### 4.1. Effects of Sprint Interval Training on EEG Results

EEG results showed that SIT was associated with higher CNS activation and enhanced neural stability after cognitive tasks. One possible interpretation, based on previous literature, is that repeated high-intensity stimulation may enhance neuroplasticity, such as increased cortical excitability and synaptic efficiency [39]. Although BDNF-related adaptations were not directly measured in the present study, prior work suggests that SIT can upregulate BDNF, which in turn may improve signal transmission and facilitate faster neural recovery [40]. This interpretation is consistent with the observed increases in CNS activation and stabilization, which may indicate improved readiness for rapid re-engagement after cognitive stress, but it remains speculative and requires direct verification. These observations should be interpreted as associations with the SIT intervention rather than evidence of direct causal effects, given the preliminary design and absence of a control group.

The longer DC stabilization time may indicate more precise, resource-intensive regulation. As CNS sensitivity increases, prolonged stabilization can help preserve signal accuracy under repeated cognitive demands. This interpretation is supported by concurrent improvements in stabilization level and curve grade, indicating better signal coherence and control [41]. These changes may also reflect enhanced top-down modulation, with frontal brain networks exerting greater control over sensorimotor systems. For skateboarders, this could mean better attentional filtering, perceptual stability, and motor planning, crucial for high-risk, real-time decision-making. However, this functional interpretation remains speculative, as we did not directly assess cortical network dynamics or task performance within this study.

While CNS activation, DC stabilization amplitude, and coherence indices improved significantly, DC tension and exhaustion levels remained unchanged after SIT. This pattern suggests that sprint interval training may boost the CNS’s functional stability and responsiveness without substantially altering its baseline excitatory state. DC tension indicates the organism’s readiness and resource mobilization, while exhaustion level reflects cumulative neurophysiological load. The lack of change in these areas suggests SIT’s short-term adaptive nature enhances neural efficiency and reactivity without altering baseline resources.

Taken together, these findings are consistent with the idea that SIT may act as a neurocognitive conditioning tool, improving energy-efficient regulation by repeatedly challenging the brain under physical stress. The improved EEG profiles are consistent with the idea that SIT can promote more stable neural regulation in elite athletes; however, the precise structural and molecular mechanisms were not measured in this study and should be investigated in future work.

### 4.2. Effects of Sprint Interval Training on HRV Results

HRV analyses suggested that SIT may enhance MF resistance by promoting a more favorable sympathetic–parasympathetic balance and greater autonomic flexibility. Key changes included a rise in LF, especially post-intervention, indicating increased sympathetic activation crucial for skateboarders during intense efforts [42]. The LF/HF ratio also rose, suggesting improved autonomic modulation for better transitions between exertion and recovery [43]. HF exhibited a U-shaped pattern, indicating initial parasympathetic withdrawal at training onset, followed by recovery and increased vagal tone later. Improvements in SDSD, PNS, and SNS showed better autonomic responsiveness, with efficient sympathetic activation and parasympathetic recovery. Changes in RP and Ti suggested enhanced recovery and stress adaptation during training. Taken together, these adjustments may indicate that SIT helps to optimize arousal–recovery regulation, benefiting skateboarders who require rapid transitions between intense efforts and brief recovery periods. [44,45]. Increased sympathetic activity aids rapid resource mobilization, while better parasympathetic rebound helps timely recovery, maintaining stability and reducing fatigue under intermittent stress [46,47].

Refined modulation of RP and Ti suggests faster recovery and improved readiness, showcasing SIT’s potential to enhance training stress tolerance and efficient recovery between sessions. This shift moves from enduring fatigue to actively managing it, maintaining control, and recovering precisely [48]. In contrast, time-domain indices such as RMSSD and SDNN did not show statistically significant main time effects, implying that baseline vagal tone and overall HRV were relatively unchanged. These indices are often considered markers of resting parasympathetic activity and general variability, rather than short-term adjustments. The lack of significant main effects for RMSSD and SDNN suggests that this 6-week SIT protocol may have influenced autonomic flexibility and transition kinetics more than resting-state stability. Based on previous studies of training-induced HRV adaptations [46,47,48], short-duration interventions may preferentially enhance the responsiveness of autonomic control, whereas more prolonged or differently structured conditioning might be required to induce robust changes in resting HRV.

It is also important to note that increases in LF and LF/HF are not uniformly beneficial and, in some contexts, may reflect heightened sympathetic dominance or cardiovascular strain rather than adaptive flexibility. In the present study, we interpreted the LF/HF pattern as functionally advantageous primarily because it co-occurred with improved recovery-related indices (e.g., higher PNS, more favorable RP, and controlled Ti) rather than with signs of autonomic exhaustion. Nonetheless, given ongoing debates surrounding LF/HF interpretation, these findings should be viewed cautiously and in conjunction with the broader HRV profile rather than in isolation.

### 4.3. Effects of Sprint Interval Training on VAS Results

The pattern of stable MF, higher ME and MO, lower PF, and concurrent improvements in EEG stability and autonomic flexibility can be interpreted using three complementary theoretical perspectives. First, the Psychomotor Efficiency Hypothesis [25] suggests that more stable cortical activation together with unchanged MF reflects a shift toward more economical neural control, allowing cognitive performance to be maintained with lower cortical interference. Second, the dissociation between higher ME and unchanged MF is consistent with the Processing Efficiency Theory [26], which differentiates performance effectiveness from the cognitive cost required to sustain it. In this context, the increase in ME may reflect greater metacognitive sensitivity rather than a true rise in cognitive strain. Third, the increase in MO and reduction in PF, accompanied by more favorable recovery-related HRV characteristics such as higher PNS and lower Ti, is compatible with the Multi-Action Plan model [27,28,29], implying a greater tendency to operate in an automatic-effective mode rather than an effortful-controlled mode.

The VAS findings are also broadly aligned with the EEG results, which showed enhanced CNS activation and stabilization, suggesting improved management of cognitive load. The rise in MO is particularly interesting. One plausible explanation is that repeated exposure to the Stroop task and the training/testing environment increased familiarity, task-specific confidence, and expectancy of improvement, thereby elevating reported motivation independently of direct changes in neurophysiological fatigue resistance. Although dopaminergic and autonomic mechanisms have been implicated in motivational processes [49], these pathways were not directly assessed in the present study, and their involvement in the current findings remains speculative. HRV results are compatible with this interpretation by showing a more favorable sympathetic–parasympathetic balance and improved recovery profiles (e.g., elevated PNS, reduced Ti), which are often associated with perceived energy, mood, and motivation.

Moreover, the reduction in PF despite persistent cognitive load implies systemic recovery improvements, likely due to enhanced circulatory efficiency and neuro-muscular resilience [47,50]. Together with the observed improvements in CNS stability and autonomic regulation, the stable subjective MF ratings suggest a coordinated multi-system adaptation, in which SIT enhances physiological readiness and neural control without increasing perceived MF fatigue under high-intensity demands.

Collectively, findings from EEG, HRV, and VAS are consistent with enhanced MF resistance potentially arising from coordinated adaptations in neural activation, autonomic regulation, and psychological resilience. This multi-system synergy reflects not only improved tolerance to MF but also a more efficient capacity to detect, manage, and recover from cognitive load—critical for sustaining high-level performance in cognitively demanding sports. The lack of change in subjective MF, despite enhanced EEG stability and autonomic flexibility, aligns with theories of neural efficiency. These frameworks propose that improved neurophysiological regulation may reduce cognitive cost without altering subjective perceptions, indicating a shift toward more automatic and resilient control modes. Importantly, the present findings do not permit causal inference. Alternative explanations, including learning effects, repeated exposure to the Stroop task, increased task familiarity, or contextual influences from the centralized training environment, cannot be excluded. These factors may have contributed to the observed neurophysiological and subjective patterns alongside the SIT intervention.

### 4.4. Limitations and Future Research

Although this study observed patterns consistent with potential benefits of SIT for MF resistance, several limitations warrant further investigation:

First, the sample size was small (*n* = 12), and the study adopted a single-group repeated-measures design within a single national-team cohort. Under the structured elite training schedule, establishing a feasible parallel control group was not realistic. Although pre- to post-intervention changes showed positive trends, this design limits the ability to eliminate potential confounders (e.g., sex differences and individual variability), and causal inference must therefore be made with caution. Future studies with larger samples and randomized controlled designs are needed to strengthen causal inference and improve generalizability.

Second, EEG and HRV indices were derived directly from the OmegaWave system, and certain algorithmic details of the proprietary processing remain undisclosed, which may affect reproducibility. Future studies should conduct independent processing of raw signals for validation and incorporate biomarkers (e.g., BDNF, lactate) and neuroimaging tools (e.g., fMRI, fNIRS) to clarify underlying neurophysiological mechanisms.

Third, the 6-week intervention and immediate post-assessment did not capture long-term effects. Because assessments were conducted only immediately after the 6-week program, the duration, decay trajectory, and minimum effective maintenance dose of SIT-induced fatigue resistance remain unclear. Future studies should employ longitudinal designs with extended follow-up and detraining-phase monitoring to determine whether neural and autonomic adaptations are sustained or reversible over time, and to establish the minimum maintenance load required. Moreover, although the 60 min Stroop paradigm we used has been widely validated as a mental-fatigue induction task, we did not analyze Stroop reaction time or accuracy in the present study, and VAS-MF scores did not show a significant increase across time points. As a result, the extent to which “classical” mental fatigue was induced in behavioral terms remains uncertain. The observed EEG and HRV adaptations should therefore be interpreted as responses to repeated cognitive load rather than definitive evidence of improved resistance to behaviorally verified mental fatigue. Future work should incorporate both Stroop performance metrics and sport-specific perceptual–motor tasks to better link neurophysiological adaptations with functional outcomes.

Finally, although the Stroop paradigm is a standardized and widely validated method for inducing mental fatigue, it does not fully reflect the perceptual-motor demands of competitive skateboarding. Future research should integrate sport-specific cognitive tasks or VR-based decision-making paradigms to enhance ecological validity. Accordingly, all findings from the present study should be interpreted as hypothesis generating rather than confirmatory, and primarily intended to inform future randomized and mechanistic investigations.

## 5. Conclusions

Within the constraints of a preliminary single-group design and the absence of a control group, this study provides exploratory evidence that a six-week sprint interval training (SIT) program may be associated with enhanced resistance to Stroop-related mental fatigue in elite skateboarders. Although behavioral performance indices were not analyzed, the observed patterns were primarily evident at the neuro-regulatory level, where central activation profiles appeared more stable, autonomic balance shifted toward a more favorable state, and subjective mental fatigue levels did not deteriorate despite repeated cognitive load. These findings should be regarded as promising but exploratory rather than definitive. Collectively, the results suggest that SIT may be linked to neural stability and autonomic modulation alongside stable subjective mental fatigue responses. As a time-efficient intervention, SIT may provide a preliminary, data-informed reference for supporting mental fatigue resistance development in this highly specialized athletic population. Future studies should expand on these findings by integrating SIT with sport-specific cognitive training tasks and controlled designs to further clarify underlying mechanisms and performance relevance.

## Figures and Tables

**Table 1:** EEG Indicators Collected by OmegaWave System.

Indicators	Definition and Interpretation
CNS Activation Level	Measures central nervous system arousal based on DC potential. Reflects the brain’s readiness and wakefulness. Optimal range indicates healthy CNS function; values below zero suggest central fatigue relative to the athlete’s individual baseline. Values are interpreted relative to manufacturer-provided normative guidelines and each athlete’s individual baseline, rather than against a fixed universal cut-off.
DC Curve Grade	Qualitative rating of the trend in physiological/psychological response (scale-based ordinal rating provided by the manufacturer). Higher grades indicate increasing activation or stress response trend.
DC Stabilization Level	Categorical assessment of how steady the DC potential signal is, indicating the stability of CNS regulation. DC Curve Grade and DC Stabilization Level are ordinal categories provided by the manufacturer; higher grades/levels indicate a more pronounced response trend and greater signal stability, respectively.
DC Stabilization (mV)	Quantitative DC potential measure (in millivolts), indicating absolute stability of the DC waveform.
DC Stabilization Time (sec)	Time required for the DC potential to reach a stable steady-state, reflecting how quickly the CNS returns to equilibrium.
DC Tension Exhaustion Level	Indicates degree of CNS depletion after prolonged activity or stress, representing both physiological and psychological resource exhaustion.

Note: CNS: Central Nervous System; DC: Direct Current potential. CNS Activation Level, DC Curve Grade, and DC Stabilization Level are interpreted according to manufacturer-derived ordinal scales and individualized baselines; exact numeric cut-offs are not publicly disclosed.

**Table 2:** HRV Indicators Collected by OmegaWave System.

Indicators	Definition and Interpretation
LF (ms^2^)	Power in the low-frequency band (0.04–0.15 Hz); reflects mixed sympathetic and parasympathetic activity, often associated with baroreflex function
HF (ms^2^)	Power in the high-frequency band (0.15–0.40 Hz); primarily indicative of parasympathetic (vagal) activity, associated with respiratory sinus arrhythmia
LF/HF	Ratio of LF to HF; commonly used to estimate sympathovagal balance, albeit with interpretation controversies
RMSSD (ms)	Root Mean Square of Successive Differences between adjacent RR intervals; sensitive to vagal modulation and widely used in short-term HRV analysis
SDNN (ms)	Standard Deviation of NN intervals; reflects overall HRV, encompassing both sympathetic and parasympathetic influences over the recording period
SDSD (ms)	Standard Deviation of Successive Differences in RR intervals; similar to RMSSD, capturing beat-to-beat variance
PNS (a.u.)	Parasympathetic Nervous System index: OmegaWave-derived score based on HRV modeling to quantify vagal tone and recovery state
SNS (a.u.)	Sympathetic Nervous System index: OmegaWave-derived score representing sympathetic activation magnitude based on HRV features
RP	Reflects daily vagal tone and recovery pattern; indicates whether cardiac autonomic state is shifting toward sympathetic or parasympathetic dominance
Ti (a.u.)	Quantifies cardiac tension or sympathetic stress based on HRV-derived features; higher values indicate elevated physiological strain

Note: LF: Low Frequency; HF: High Frequency; LF/HF: Low Frequency to High Frequency Ratio; RMSSD: Root Mean Square of Successive Differences; SDNN: Standard Deviation of Normal to Normal intervals; SDSD: Standard Deviation of Successive Differences; PNS: Parasympathetic Nervous System index; SNS: Sympathetic Nervous System index; RP: Recovery Pattern; Ti: Tension Index; HRV: Heart Rate Variability; RR: R–R Interval; NN: Normal-to-Normal Interval.

**Table 3:** Descriptive statistics of the EEG parameters in pre-intervention, mid-intervention, and post-intervention.

Variable	Pre-Intervention Mean ± SD (Min–Max)	Mid-Intervention Mean ± SD (Min–Max)	Post-Intervention Mean ± SD (Min–Max)	*F*/*χ*^2^	*p*	*η*^2^/W
CNS Activation Level	0.63 ± 5.89 ^bc^ (−8.37–9.32)	7.34 ± 9.39 ^a^ (−8.64–24.19)	8.66 ± 10.31 ^a^ (−6.75–29.54)	6.839	0.018 *	0.383
DC Curve Grade	3.68 ± 1.04 (2.60–6.65)	3.61 ± 1.52 ^c^ (1.20–6.86)	4.78 ± 1.95 ^b^ (1.47–8.73)	10.667	0.005 **	0.444
DC Stabilization Level	3.50 ± 1.07 ^bc^ (1.99–5.41)	4.51 ± 1.47 ^ac^ (1.96–6.01)	5.44 ± 1.86 ^ab^ (2.40–8.57)	12.211	0.003 **	0.526
DC Stabilization (mV)	0.19 ± 6.09 ^c^ (−8.14–10.01)	7.01 ± 9.29 (−8.54–24.18)	8.63 ± 10.50 ^a^ (−6.71–29.53)	12.167	0.002 **	0.507
DC Stabilization Time (sec)	107.85 ± 1.02 ^c^ (104.62–108.15)	110.58 ± 5.82 ^c^ (104.28–121.11)	134.86 ± 7.86 ^ab^ (126.73–150.14)	19.478	0.000 **	0.812
DC Tension Exhaustion Level	−2.65 ± 1.10 (−4.09–−0.93)	−1.90 ± 1.20 (−4.14–0.03)	−2.43 ± 1.53 (−5.05–0.04)	5.167	0.076	0.215

Note: a, b, and c indicate significant differences compared to pre-intervention, post-intervention, and mid-intervention, respectively; *p* < 0.05 is considered statistically significant (*), and *p* < 0.01 denotes highly significant differences (**). Effect size is reported as partial *η*^2^ for variables analyzed with repeated-measures ANOVA and as Kendall’s *W* for variables analyzed with the Friedman test.

**Table 4:** Descriptive statistics of the HRV parameters in pre-intervention, mid-intervention, and post-intervention.

Variable	Pre-Intervention Mean ± SD (Min–Max)	Mid-Intervention Mean ± SD (Min–Max)	Post-Intervention Mean ± SD (Min–Max)	*F*/*χ*^2^	*p*	*η*^2^/W
LF (ms^2^)	585.35 ± 524.12 (34.33–1987.64)	609.36 ± 997.34 ^c^ (55.25–3397.04)	1051.17 ± 1413.01 ^b^ (67.61–4114.50)	8.167	0.017 *	0.340
HF (ms^2^)	1201.09 ± 988.92 ^b^ (93.47–3561.06)	966.57 ± 1361.82 ^ac^ (32.46–4787.21)	1211.65 ± 1653.00 ^b^ (40.00–5852.43)	8.000	0.018 *	0.333
LF/HF	0.76 ± 0.84 ^c^ (0.18–2.69)	1.15 ± 1.13 ^c^ (0.08–3.31)	1.45 ± 1.37 ^ab^ (0.10–4.04)	10.500	0.005 **	0.438
RMSSD (ms)	83.55 ± 31.75 (46.86–163.66)	83.39 ± 33.72 (43.69–169.97)	100.11 ± 41.94 (53.30–208.36)	5.167	0.076	0.215
SDNN (ms)	73.41 ± 23.51 (31.20–117.61)	67.35 ± 30.79 ^c^ (29.26–136.01)	83.03 ± 37.71 ^a^ (35.69–166.90)	3.270	0.096	0.229
SDSD (ms)	108.76 ± 41.09 (60.39–215.24)	107.36 ± 41.90 ^c^ (58.36–216.44)	140.87 ± 51.42 ^b^ (71.27–264.31)	11.167	0.004 **	0.465
PNS (a.u.)	0.54 ± 0.15 ^c^ (0.25–0.75)	0.51 ± 0.18 ^c^ (0.27–0.86)	0.65 ± 0.22 ^ab^ (0.33–1.05)	8.200	0.010 *	0.427
SNS (a.u.)	0.44 ± 0.10 ^c^ (0.32–0.67)	0.47 ± 0.10 ^c^ (0.30–0.65)	0.60 ± 0.12 ^ab^ (0.30–0.79)	20.468	0.000 **	0.853
RP	0.31 ± 0.09 (0.13–0.44)	0.29 ± 0.11 ^c^ (0.14–0.51)	0.37 ± 0.14 ^b^ (0.17–0.63)	5.957	0.029 *	0.351
Ti (a.u.)	68 ± 62 ^c^ (19–250)	73 ± 56 ^c^ (12–223)	90 ± 69 ^ab^ (15–273)	12.667	0.002 **	0.528

Note: a, b, and c indicate significant differences compared to pre-intervention, post-intervention, and mid-intervention, respectively; *p* < 0.05 is considered statistically significant (*), and *p* < 0.01 denotes highly significant differences (**). Effect size is reported as partial *η*^2^ for variables analyzed with repeated-measures ANOVA and as Kendall’s W for variables analyzed with the Friedman test.

**Table 5:** Descriptive statistics of the VAS parameters in pre-intervention, mid-intervention, and post-intervention.

Variable	Pre-Intervention Mean ± SD (Min–Max)	Mid-Intervention Mean ± SD (Min–Max)	Post-Intervention Mean ± SD (Min–Max)	*F*	*p*	*η* ^2^
MF	36.06 ± 5.04 (30.08–44.31)	37.08 ± 3.96 (31.75–45.47)	37.51 ± 5.71 (28.46–47.70)	1.156	0.333	0.095
ME	45.56 ± 10.69 ^bc^ (27.85–60.17)	49.48 ± 13.46 ^ac^ (28.35–68.81)	59.73 ± 8.24 ^ac^ (45.63–71.25)	39.759	0.000 **	0.783
MO	−11.95 ± 1.89 ^bc^ (−14.56–−8.42)	−6.94 ± 2.24 ^ac^ (−11.43–−3.05)	−0.04 ± 1.72 ^ac^ (−2.79–2.24)	145.617	0.000 **	0.930
PF	4.02 ± 1.63 ^c^ (1.35–6.62)	3.48 ± 1.46 (1.47–6.14)	2.58 ± 0.68 ^a^ (1.81–3.87)	3.494	0.048 *	0.241

Note: a, b, and c indicate significant differences compared to pre-intervention, post-intervention, and mid-intervention, respectively; *p* < 0.05 is considered statistically significant (*), and *p* < 0.01 denotes highly significant differences (**).

## Data Availability

The original data presented in the study are openly available in the supplementary materials.

## Supplementary Materials

The following supporting information can be downloaded at: https://www.mdpi.com/article/10.3390/life16010025/s1.

## Abbreviations

The following abbreviations are used in this manuscript:

SIT	Sprint Interval Training
MF	Mental Fatigue
EEG	Electroencephalography
HRV	Heart Rate Variability
VAS	Visual Analogue Scale
CNS	Central Nervous System
HIIT	High-Intensity Interval Training
HRmax	Maximal Heart Rate
RPE	Rating of Perceived Exertion
SDNN	Standard Deviation of Normal-to-Normal Intervals
RMSSD	Root Mean Square of Successive Differences
ANS	Autonomic Nervous System
PNS	Parasympathetic Nervous System
SNS	Sympathetic Nervous System
Ti	Tension Index
ME	Mental Exertion
MO	Motivation
PF	Physical Fatigue
RP	Recovery Pattern
